# Strength of selection in lung tumors correlates with clinical features better than tumor mutation burden

**DOI:** 10.1038/s41598-024-63468-z

**Published:** 2024-06-03

**Authors:** Ivan P. Gorlov, Olga Y. Gorlova, Spyridon Tsavachidis, Christopher I. Amos

**Affiliations:** Institute for Clinical and Translational Research, Baylor College of Medicine, One Baylor Plaza, Mailstop: BCM451, Houston, TX 77030 USA

**Keywords:** Cancer, Evolution

## Abstract

Single nucleotide substitutions are the most common type of somatic mutations in cancer genome. The goal of this study was to use publicly available somatic mutation data to quantify negative and positive selection in individual lung tumors and test how strength of directional and absolute selection is associated with clinical features. The analysis found a significant variation in strength of selection (both negative and positive) among tumors, with median selection tending to be negative even though tumors with strong positive selection also exist. Strength of selection estimated as the density of missense mutations relative to the density of silent mutations showed only a weak correlation with tumor mutation burden. In the “all histology together” analysis we found that absolute strength of selection was strongly correlated with all clinically relevant features analyzed. In histology-stratified analysis selection was strongest in small cell lung cancer. Selection in adenocarcinoma was somewhat higher compared to squamous cell carcinoma. The study suggests that somatic mutation- based quantifying of directional and absolute selection in individual tumors can be a useful biomarker of tumor aggressiveness.

## Introduction

Single nucleotide substitutions (SNSs) are the major type of somatic variation in tumors^1,2^. Even though the absolute majority of the point mutations are neutral^3^, there are many examples of positive and negative selection of point mutations in carcinogenesis^4,5^. The absolute majority of the analyses of selection in tumors has been done at the level of individual genes^5–7^, while a quantitative assessment of the direction and strength of selection at the tumor level has never been addressed according to our best knowledge.

Quantifying of the strength of selection at the tumor level can be used for a better understanding of tumor biology and can reflect tumor aggressiveness because quickly evolving tumors can better adapt to the host immune response and chemotherapy, and as a result, survive better and proliferate more quickly^8^.

The most commonly used global somatic mutation-based biomarker is tumor mutational burden (TMB). TMB is a tumor feature that predicts survival^9^, risk of metastasis, progression^10,11^, and response to treatment, especially to immunotherapy^12–14^. We hypothesized that aside from TMB, strength of selection in a tumor may also reflect the speed of tumor evolution and therefore can be associated with clinically relevant features.

We hypothesized that assessment of the global selection based on somatic mutations in a tumor is associated with clinical features and potentially could be used as a biomarker of cancer aggressiveness. Tumor development is an evolutionary process comprising differential survival and proliferation of genetically different cell lineages (clones)^15,16^. Cancer cells that survive better and proliferate faster have a selective advantage and over time become a predominant clone of genetically heterogeneous tumor^17^. This evolution happens even before any treatment is applied, though treatment itself is a very strong selective factor that drives tumor evolution^18^.

Individual tumors as well as clones inside a tumor differ by their intrinsic propensity to produce somatic mutations, which depends on their DNA repair capacity as well as environmental exposures which is especially relevant to lung cancer^19,20^. These factors contribute to the tumor’s ability to evolve through Darwinian selection. Fast-evolving tumors tend to be more aggressive since they better adapt to the host immune response and proliferate more quickly compared to slowly evolving tumors^21^.

The direction and strength of selection in coding regions of the human genome can be quantified by the ratio of substitution rates at non-synonymous and synonymous sites, *dN/dS*. Even though it is not perfect^22^, this metric is widely used across different research fields^23^. The measure was first based on the comparison of homologous sequences to estimate selection strength in species divergence, and recently the approach became a popular tool to quantify selection strength in tumor^4,24,25^. Nonsynonymous to synonymous mutation ratio was used to estimate strength of selection in individual genes across cancer types^26–28^. A study by Persi et al.^29^ used dN/dS ratio for a pan-cancer analysis of selection in 6,721 tumors representing 23 cancer types. They found that strength of selection in tumor is associated with tumor fitness and found “likely clinical implications” of *dN/dS*.

The goal of our study is to quantify global selection in individual lung tumors and to test if the strength of selection is clinically relevant. Clinical relevance is assessed by the analysis of correlation of the strength of positive, negative and absolute selection in individual lung tumors with clinical features.

## Methods

### Description of the approach

We have estimated the direction (negative or positive) and strength of selection by a comparison of the densities of nonsynonymous (missense) to synonymous (silent) mutations. In this respect our approach is similar to the commonly used ratio on nonsynonymous to synonymous substitutions *dN/dS*^22,30,31^. Our approach, however, differs from *dN/dS* method in the manner of how the normalization of the mutation numbers is done. Our goal was to quantify the strength of positive and negative selection at genome level while *dN/dS* estimates are designed for an assessment of selection in individual genes. At the genome level, exactly the same single nucleotide substitution may produce nonsynonymous or synonymous mutation depending on what transcript is considered. This ambiguity stems from the fact that the absolute majority of the genes in the human genome undergo alternative splicing^32^. Together with the common (up to a quarter of all genes) cases of overlapping genes^33^ this leads to a quite common situation when the same nucleotide substitution results in either a nonsynonymous or a synonymous substitution depending on what transcript is analyzed. The key parameter in our analysis is the number of potential sites for missense and silent mutations in the human genome. To estimate the number of potential sites for silent and missense mutations in the human genome we computationally “mutated” each nucleotide in coding regions into 3 possible single nucleotide substitutions and ran the “mutated” sequence against all known transcripts to see if it produced a silent or a missense mutation^34^. Therefore, we counted the total number of missense and silent mutations that can be produced by all possible single nucleotide substitutions in the human genome in the context of all existing transcripts. This approach fits well with how somatic mutations are reported in the Catalog Of Somatic Mutations In Cancer (COSMIC) database which we have used as the data source for the study. In COSMIC the same point mutation may be reported as missense or silent depending on the transcript. The other difference of our approach from *dN/dS* method was that we have used the logarithm of the ratio instead of the simple ratio of nonsynonymous to synonymous mutations. This was done to make the distribution more symmetrical and therefore more suitable for statistical comparisons.

### Estimation of the number of potential sites in the human genome for missense and silent mutations

We used the latest build of the human genome project—GRCh38 to estimate the number of potential sites for missense and silent mutations. We first identified all nucleotide positions in the consensus protein coding sequence (CCDS) database^35^. Then we computationally mutated each nucleotide into the three possible single nucleotide substitutions (SNSs) and checked if a given SNS led to a missense or a silent mutation. If the SNS produced both missense and silent mutations it was counted both ways: as a potential site for both missense and silent mutations. This way we have estimated the total number of potential sites for missense and nonsense mutations in the human genome to be equal to 74,038,110 and the total number of potential sites for silent mutations to be 22,654,380.

### Quantifying of negative and positive selection

The number of missense mutations in a tumor can be used to detect the type of selection (negative or positive) and to quantify the strength of selection. Negative selection against missense mutations will result in their lower number while positive selection will increase their number, and both affect the missense mutation density. However, selection is not the only factor influencing the number of somatic mutations in tumor. Environmental exposures, for example, tobacco smoke, dramatically increase the number of somatic mutation in lung tumors^36^. One needs to take into account the overall mutability when estimating direction and strength of selection of missense mutations. Silent mutations can be used to adjust for tumor-specific mutability. Despite anecdotal examples of functionality^37,38^, silent mutations are generally selectively neutral^39^ and, therefore, silent mutations can be used as a reference group.

As a measure of selection we used the logarithm of the ratio of the densities of missense to silent mutations, that is, the number of missense mutations per million of potential sites to the number of silent mutations per million of potential sites. Negative log ratio values of relative selection indicate selection against missense mutations (negative selection), and positive log ratios indicate positive selection for missense mutations. To estimate strength of selection regardless of its direction we used the absolute value of the log ratio. We also estimated tumor mutation burden (TMB) for each tumor. TMB was defined as the number of missense mutations detected in a given tumor by whole exome sequencing.

### Somatic mutation data

We used somatic mutation data from the Catalog Of Somatic Mutations In Cancer (COSMIC)^40^. COSMIC is the largest repository of somatic mutations detected in tumor samples. COSMIC is updated quarterly, with the sample size increasing 5–10% with each new version. We used the latest version (V98) of the database. We focused on lung cancer because it has one of the highest numbers of reported somatic mutations compared to other cancers^41–43^. The summary of the data used in this study can be found in Supplementary Table S1.

We tested the association of (1) strength of positive selection, (2) strength of absolute selection regardless of the direction, and (3) tumor mutation burden with three clinically relevant tumor features: tumor stage, patient age at diagnosis and a comparison between primary and metastatic tumors. We used the Spearman rank-order correlation coefficient (rho) to test the association of selection strength with age at diagnosis. To test the association between selection and tumor stage we used nonparametric Spearman's rank correlation coefficient, and t-test to compare strength of selection between primary and metastatic tumors. The clinical characteristics were downloaded from COSMIC website. Stage information was available for 26% of tumors, the age at diagnosis for 85% of all patients, and primary (82%) versus metastatic (18%) for 60% of COSMIC samples .

### Analysis of global selection in lung tumors stratified by the presence of driver mutations in EGFR or KRAS

We stratified tumor samples by the presence/absence of driver mutations in EGFR and KRAS genes. We used these genes because the largest number of samples harbored driver mutations in them: 69 samples with an EGFR driver mutation and 160 samples with a KRAS driver mutation. For EGFR we considered as a driver any of the following COSMIC reported mutations: p.L858R, p.L813R, p.T790M, p.T745M, p.R521K, and p.R476K^44^. For KRAS the following COSMIC reported mutations were considered as drivers: p.G12C, p.G12V, p.G12D, p.G12A, p.G13C, p.G12S, and p.G13D^45^.

## Results

### Quantifying selection in individual tumors: joint analysis of all cell types

**Figure 1:**
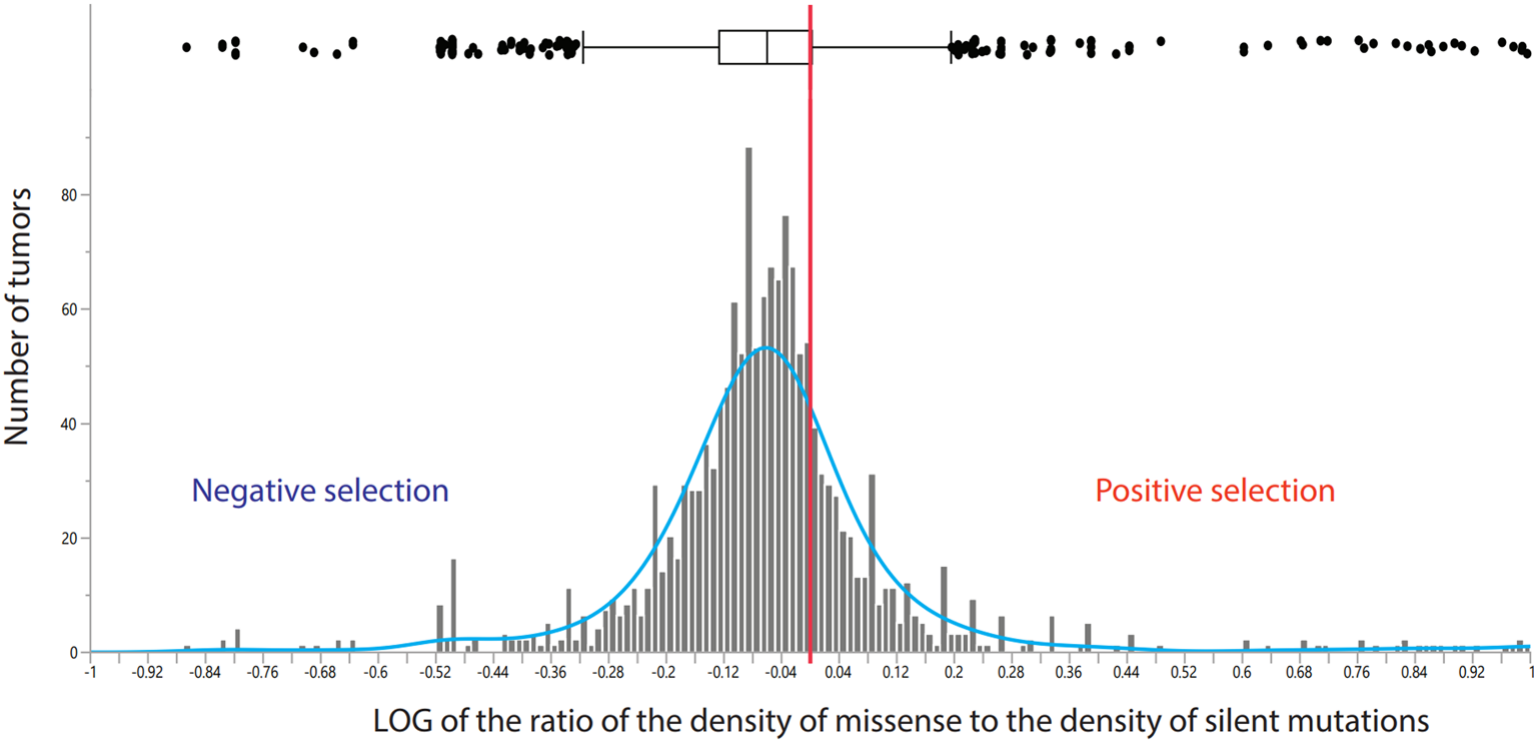
The distribution of the log ratio of the density of missense to the density of silent mutations. The vertical red line marks the relative density expected when the global selection is zero, that is, the density of missense mutations equals the density of silent mutations. The median log ratio is shown as a vertical line on the box plot. The standard deviation SD = 0.268 is shown as a horizontal box. Vertical bars show the 95% confidence interval.

Figure 1 shows the distribution of log ratios of the density of missense over the density of silent mutations in individual lung tumors. Denote MmD as the missense mutation density estimated as the number of missense mutation per million of potential sites for missense mutations, and SmD, the silent mutation density, as the number of silent mutations per million of potential sites for silent mutations. We found that the mean log(MmD/SmD) in all cell types analyzed together was equal to -0.034 ± 0.007. Single sample t-test against mean log(MmD/SmD) = 0 (no selection) was -5.1, which is highly statistically significant with p = 7 × 10^−7^. The result indicates that global selection on missense mutations in lung tumors is negative.

### The association of global tumor selection with clinically relevant features.

Table 1 shows the results of statistical analyses of the association of directional and absolute selection with clinically relevant features available. The table also shows the association between clinically relevant features and tumor mutation burden. The total number of missense mutations detected in a given tumor was used as TMB. Directional selection was significantly associated with age at diagnosis and primary versus metastatic tumors. Absolute global selection was significantly associated with all clinically relevant features while TMB does not show any significant association with clinically relevant features.

**Table 1:** Strength of the statistical association of clinically relevant characteristics with the strength of directional selection (expressed as log(MmD/SmD)), the strength of absolute selection (expressed as its absolute value ABS{log(MmD/SmD)}), and with tumor mutation burden.

Predictor	Clinically relevant feature		
	Age at diagnosis	Stage	Metastatic versus primary
Directional selection	**rho = − 0.07, N = 1.565, p = 0.007**	Spearman R = − 0.01, N = 309, p = 0.91	**(0.582 + − 0.086 versus − 0.052 + − 0.007) t-test = 17.77, df = 912, p < 10^ − 12**
Absolute selection regardless of direction	**rho = − 0.10, N = 1.565, p = 0.00007**	**Spearman R = 0.14, N = 309, p = 0.01**	**(0.726 + − 0.059 versus 0.13 + − 0.006) t-test = 21.66, df = 912, p < 10^ − 24**
Tumor mutation burden (TMB)	rho = − 0.05, N = 1.885, p = 0.05	Spearman R = − 0.06, N = 500, p = 0.15	(128.5 + − 18.9 versus 121.3 + − 5.4) t-test = 0.33, df = 1,137, p = 0.74

Significant values are in [bold].

### The shape of the association of directional selection with clinically relevant features

To study the shape of the associations between clinically relevant features and selection we have stratified all tumors into five categories based on the selection strength: (1) *strong negative selection*—log ratio < -0.2 (total 189 tumors), (2) *weak negative selection,* -0.02 ≤ log ratio < -0.05 (total 661 tumors), (3) *no obvious selection,* -0.05 ≤ log ratio < 0.05 (total 461 tumors), (4) *weak positive selection,* 0.05 ≤ log ratio < 0.2 (total 157 tumors), and (5) *strong positive selection,* log ratio ≥ 0.2 (total 97 tumors). The categorization was based on the following considerations: (i) to facilitate the interpretation, categories needed to be distributed symmetrically relative to zero (as zero means no selection); (ii) the categories were made maximally similar in size, to make the comparisons more robust. That was not a simple task because the whole distribution is shifted to the left relative to zero.

**Figure 2:**
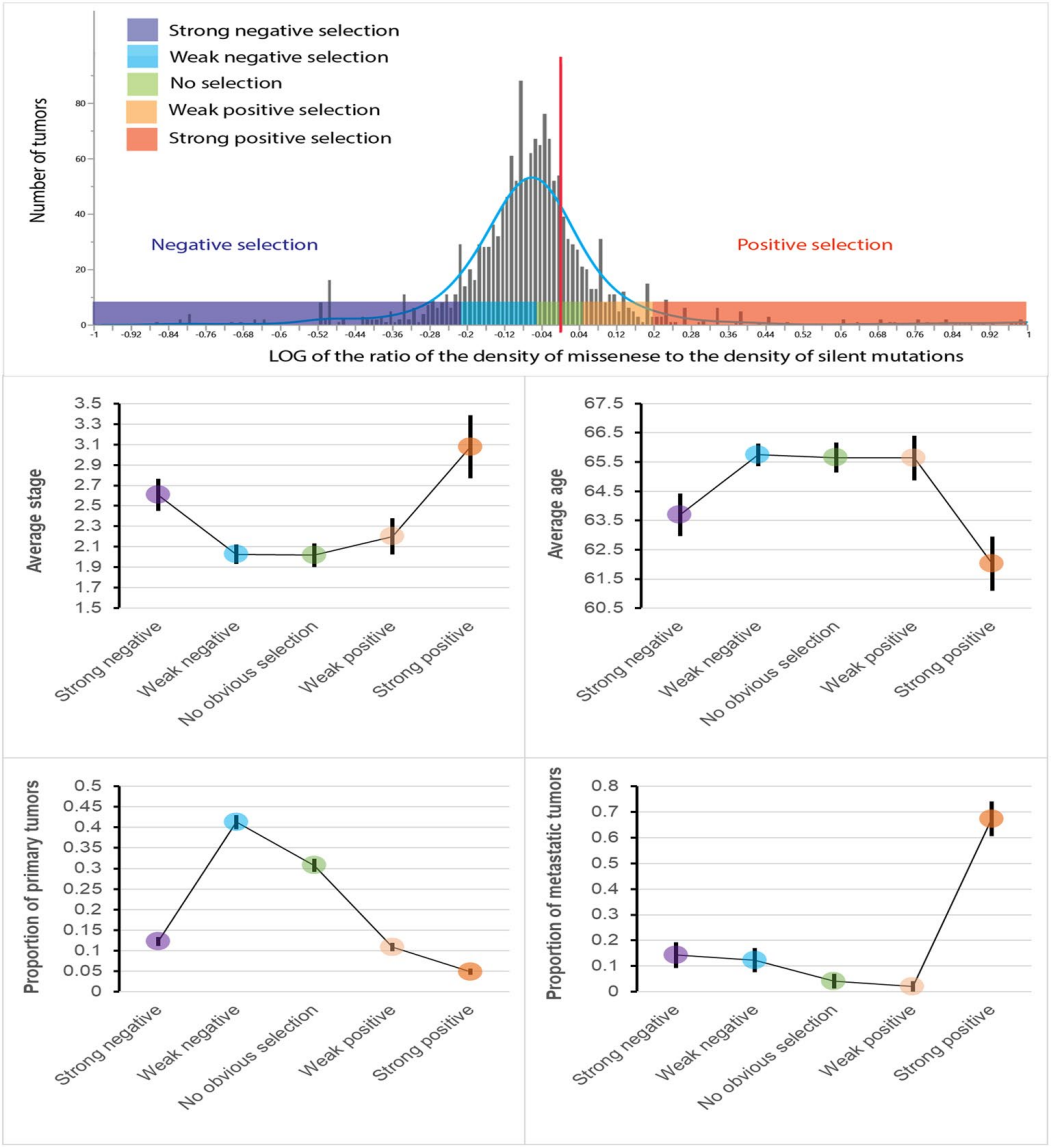
Upper panel shows stratification of tumors into five categories of strength of directional selection. Vertical red line marks the point of zero global selection. The middle and low rows show distributions of values of clinically relevant features in tumors categorized by strength of the directional selection. Note U-shaped or reverse U-shaped associations with the clinically relevant features.

The upper panel of the Fig. 2 shows the positions of the five categories (colored boxes) relative to the distribution of the strength of directional selection. The four lower panels of the Fig. 2 show the results of the analysis. For the study of the shape of association between directional selection and stage (second row, left panel), stage was treated as ordered numbers reflecting tumor progression, with Stage I being least and Stage IV most advanced. For all analyzed clinically relevant traits we observed a U-shaped or an inverse U-shaped association between directional selection and the analyzed features. The results indicate that the absolute strength of the selection rather than the direction of selection is clinically relevant.

### Histology-specific analysis of the global selection in lung tumors

**Figure 3:**
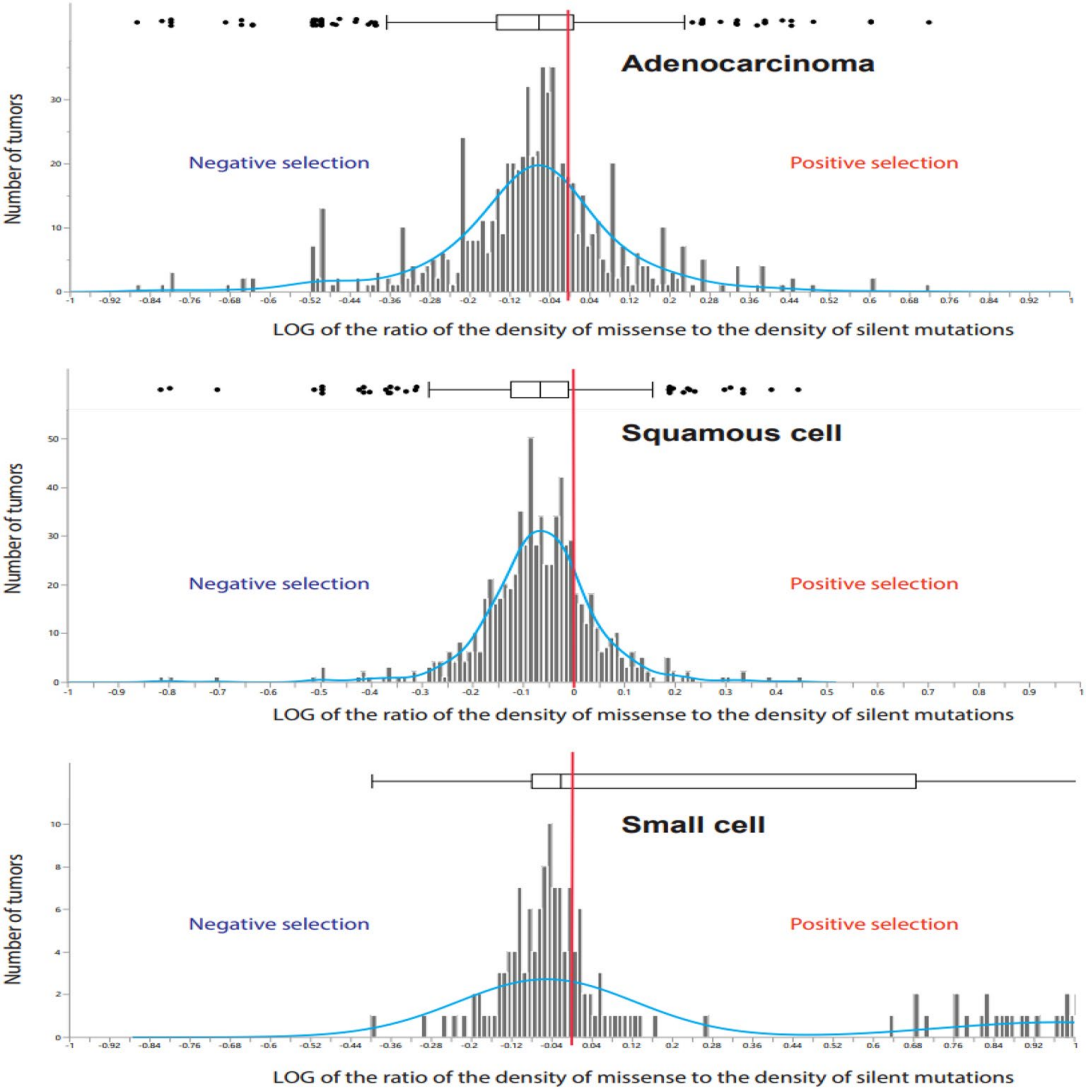
The distribution of the ratio of the density of missense to the density of silent mutations in adenocarcinoma (top panel), squamous cell carcinoma (middle panel), and small cell lung cancer (lower panel). The vertical red line marks the relative density expected in the absence of selection. Median log ratio is shown as the vertical line on the box plot.

Figure 3 shows the distributions of log(MmD/SmD) in three major lung cancer cell types: adenocarcinoma—685 tumors, squamous cell carcinoma—713 tumors, and small cell lung cancer—167 tumors. In all cell types combined the mean log ratio was lower than zero, indicating global negative selection. The mean log ratio for adenocarcinoma was -0.06 ± 0.01 which is significantly lower than zero: t = 5.9, p = 6.1 × 10^−9^. For squamous cell carcinoma the mean log ratio was -0.070 ± 0.005, t = 15.1, p < 10^−24^, and for small cell lung cancer the mean ratio was positive: 0.23 ± 0.04, t-test = 6.0, p = 10^−8^. The positive mean global selection in small cell lung cancer is due to the presence of a cluster of tumors with strong positive selection (see the far right part of the distribution). However, the median value of the log ratio for small cell lung cancer was negative − 0.02, along with the median values for adenocarcinoma and squamous cell carcinoma, -0.06 and -0.07, correspondingly.

### Association of global tumor selection with three clinically relevant features: histology specific analysis

Table 2 shows the results of the statistical analysis of the associations between selection and clinically relevant features in analyses stratified by histology. The absolute selection shows four significant associations across histology. TMB has three and directional selection—two significant associations with clinically relevant characteristics.

**Table 2:** Strength of the statistical association between directional selection, absolute selection and tumor mutation burden with clinically relevant characteristics.

Predictor	Clinically relevant feature		
	Age at diagnosis	Stage	Metastatic versus primary
ADENOCARCINOMA			
Directional selection	rho = − 0.02, *N* = 685, *p* = 0.52	Spearman R = − 0.14, *N* = 69, *p* = 0.92	**(**− **0.067 ± 0.010 versus **− **0.314 ± 0.089) *t*-test = 3.2, df = 474, *p* = 0.001**
Absolute selection regardless of direction	**rho = **− **0.11, *N* = 685, *p* = 0.003**	Spearman R = − 0.20, *N* = 69, *p* = 0.11	**(0.153 ± 0.008 versus 0.326 ± 0.083) *t*-test = 2.9, df = 474, *p* = 0.004**
Tumor mutation burden	**rho = 0.15, *N* = 925, *p* = 0.0002**	rho = − 0.06, *N* = 242, *p* = 0.38	(91.1 ± 7.7 versus 38.3 ± 22.4) *t*-test = 1.5, df = 1,137, *p* = 0.15
SQUAMOUS CELL CARCINOMA			
Directional selection	rho = − 0.04, *N* = 713, *p* = 0.35	Spearman R = − 0.13, *N* = 129, *p* = 0.14	NA
Absolute selection regardless of direction	rho = − 0.01, *N* = 713, *p* = 0.85	**Spearman R = **− **0.43, *N* = 129, *p* = 0.0001**	NA
Tumor mutation burden	rho = − 0.07, *N* = 747, *p* = 0.06	**Spearman R = **− **0.39, *N* = 747, *p* = 0.0004**	NA
SMALL CELL LUNG CANCER			
Directional selection	rho = − 0.11, *N* = 167, *p* = 0.16	Spearman R = − 0.06, *N* = 111, *p* = 0.51	**(0.031 ± 0.031 versus **− **0.757 ± 0.076) *t*-test = 11.1, df = 912, p < 10**^12^
Absolute selection regardless of direction	rho = − 0.08, *N* = 167, *p* = 0.28	Spearman R = − 0.11, *N* = 111, *p* = 0.91	**(0.146 ± 0.025 versus 0.806 ± 0.062) *t*-test = 11.7, df = 912, p < 10**^12^
Tumor mutation burden	**rho = **− **0.20, *N* = 213, *p* = 0.003**	Spearman R = − 0.05, *N* = 114, *p* = 0.59	(178.4 ± 10.9 versus 186.7 ± 24.1) *t*-test = 0.35, df = 1,137, *p* = 0.73

NA—there were no samples from metastatic sites for squamous cell carcinoma.

Significant values are in [bold].

## Analysis of the strength of global selection in lung tumors stratified by the presence of common driver mutations

Table 3 describes the results of the analysis of the strength of selection in lung tumors stratified by presence/absence of driver mutations in EGFR and KRAS. Log(dN/dS) in tumor samples with EGFR driver mutations was -0.13 ± 0.01 which is significantly lower compared to the strength of global selection in samples without EGFR driver mutations − 0.06 ± 0.01; t-test = 4.54, p = 3.1 × 10^−6^. For KRAS we observed the opposite difference: log(dN/dS) for samples with KRAS driver mutations was 0.01 ± 0.01 and for samples without a KRAS driver mutation − 0.06 ± 0.01 : t-test = 5.81, p = 2.5 × 10^−11^. The differences in the direction of the effect can be related to the fact that EGFR is an oncogene^46^ and wild type KRAS is a wild type tumor suppressor^47^ (see Discussion section for details).

**Table 3:** Missense and silent mutations in samples categorized by the presence of driver mutations in EGFR and KRAS genes.

Sample type	Number of samples	Number of missense mutations	Number of silent mutations	Density missense (per sample, per site)	Density silent (per sample, per site)	dN/dS
Driver mutations in EGFR	69	14,700	6035	2.88E-06	3.86E-06	0.75 ± 0.03
No driver mutations in EGFR	2362	855,254	291,208	4.89E-06	5.44E-06	0.89 ± 0.01
Driver mutations in KRAS	160	80,537	24,213	6.80E-06	6.68E-06	1.02 ± 0.02
No driver mutations in KRAS	2271	789,200	272,442	4.69E-06	5.30E-06	0.89 ± 0.01

## Discussion

Somatic mutations play an important role in cancer development^48–50^. Missense and silent mutations are the two most common types of somatic mutations. Though most missense mutations are neutral^48–51^, some of them are functional and play an important role in tumorigenesis^52,53^. As for silent mutations, the absolute majority of them are neutral, with only rare examples of functionality^54^, and for this reason they can be used as a reference group to quantify strength and direction of selection on missense mutations. If the density of missense mutations in a tumor is lower compared to the density of silent mutations, the global selection is negative. Conversely, if the density of missense mutations is higher than the density of silent mutations, the global selection is positive.

Strength of selection is an indicator of how quickly a tumor evolves: tumors with signs of strong selection evolve more quickly compared to the tumors that do not show signs of strong selection^4,55,56^. Quickly evolving tumors better survive and propagate faster and generally tend to be more aggressive compared to slower evolving tumors^15,57^. It is important, therefore, to quantify strength of global selection in individual tumors as a potential biomarker of tumor aggressiveness. The goal of this study is to introduce somatic mutation-based quantitative measure of selection in individual tumors and provide its initial validation as a biomarker of tumor aggressiveness. We also compared strength of selection (both directional and absolute) with tumor mutation burden by studying their associations with select clinically relevant characteristics.

We found that lung tumors are very diverse in terms of strength and direction of selection. In the “all histology together” analysis we found that the average global selection is negative; however, some tumors bear strong signs of positive selection. We used three clinically relevant features available from COSMIC database: clinical stage, age at diagnosis, and source of the tumor tissue (primary versus metastatic) which can be useful to assess the role of selection in metastasizing. We found that in the “all histology together” analysis all clinically relevant features show U-shaped or inverse U-shaped associations with the directional selection. This observation suggests that absolute selection will be a better predictor of clinically relevant features than directional selection. This is exactly what we have found (Table 1).

Many studies have been published on the utility of somatic mutations as predictors of tumor progression, recurrence, metastasizing and response to treatment^58–62^. Tumor mutation burden is the most commonly used somatic mutation-derived biomarker^63^. TMB is associated with survival and response to treatment in many cancer types including lung cancer^64–66^. The goal of our study was to define global absolute selection in individual tumors and introduce it as a potential biomarker that is different from TMB. One of the drawbacks of TMB is that it depends not only on strength of selection but also on the overall mutability of the tumor. We take into account the overall mutability by using the ratio of the density of missense to the density of silent mutations. We believe that the absolute logarithm of the ratio of mutation densities better reflects strength of selection than TMB does. The results of this analysis indicate that the absolute selection may be a complementary biomarker of cancer aggressiveness to TMB. Even though we found a significant positive correlation between TMB and directional selection, the correlation was relatively small: rho = 0.12, n = 1.565, p = 0.00002. The correlation between TMB and absolute (non-directional) strength of selection was also significant but negative: rho = -0.06, n = 1.565, p = 0.01. These results suggest that global selection in tumor can be used as an independent predictor of cancer aggressiveness. The relative utility of TMB and strength of selection as biomarkers is a topic of future studies.

Histology-stratified analysis of selection demonstrated significant differences in selection among the three major lung cancer cell types. The cell types differ by absolute strength of selection, with squamous cell carcinoma showing the weakest, adenocarcinoma showing intermediate, and small cell carcinoma—the strongest absolute selection. Interestingly, the variation in absolute strength of selection followed aggressiveness, with squamous cell carcinoma considered to be slow growing and the least aggressive form of lung cancer^67^, small cell lung cancer considered most aggressive^68^, and adenocarcinoma showing intermediate aggressiveness^69^. This supports the idea that absolute strength of selection in tumor can be an indicator of tumor aggressiveness.

One of the possible reasons why absolute strength of global selection in tumor can be a better biomarker compared to tumor mutational burden is its dependency of copy number variation (CNV). CNVs, especially those involving whole chromosomes and large chromosomal regions, directly influence the total number of somatic mutations and, as a result, directly influence TMB. Since estimates of global selection in tumor are based on the ratio of non-synonymous to synonymous substitutions, the measure is less sensitive to the copy number variation than TMB and therefore may be more reliable.

We found that the presence of driver mutations in lung tumors was associated with significant changes in the strength of the global selection, which is not surprising taking into account the profound effect of driver mutations on clonal evolution and tumor growth rate^70,71^. Interestingly, oncogenic driver mutations in EGFR are associated with more negative while oncogenic driver mutations in KRAS are associated with more positive selection. This can be explained by different effects of EGFR and KRAS driver mutations on DNA repair. Driver mutations in EGFR are associated with decreased DNA repair capacity in non‐small cell lung cancer^72^. KRAS driver mutations, on the other hand, are associated with more efficient DNA repair in lung tumors^73^ which may contribute to poor response of KRAS driver mutation-positive tumors to radiotherapy^74^. Since the absolute majority of de novo mutations tend to have negative effect on fitness at both the population^75^ and cellular levels^76^, one can expect that higher mutability associated with EGFR drivers will result in stronger negative selection, while improved DNA repair associated with KRAS drivers will have an opposite effect: reduced negative selection as it was observed in this study.

## Conclusion

To conclude, we propose to use the absolute value of the logarithm of relative densities of missense to silent mutations as a quantitative measure of selection in tumor. We hypothesize that the strength of absolute selection reflects tumor aggressiveness and may be used as a biomarker of tumor aggressiveness.

### Supplementary Information

Supplementary Table S1.

## Data Availability

All data generated or analyzed during this study are included in this published article (and its supplementary information files). The corresponding author will share any additional relevant data upon request (ivan.gorlov@bcm.edu).
